# Supplementary material for: Synthesis and Biological Evaluation of Amino Chalcone Derivatives as Antiproliferative Agents
Source: Molecules. 2020 Nov 25;25(23):5530. doi: 10.3390/molecules25235530 (PMC7728372; doi:10.3390/molecules25235530)
Supplement: Supplementary file 1 [file molecules-25-05530-s001.pdf]

Synthesis and biological evaluation of amino chalcone derivatives as antiproliferative agents

Chao-Fan Lu [1,†], Sheng-Hui Wang [1,†], Xiao-Jing Pang [1,2,†], Ting Zhu [2], Hong-Li Li [1], Qing-Rong Li [1], Qian-Yu Li [1], Yu-Fan Gu [1], Zhao-Yang Mu [1], Min-Jie Jin [1], Yin-Ru Li [1], Yang-Yang Hu [4], Yan-Bing Zhang [2], Jian Song [1,2,*] and Sai-Yang Zhang [1,2,3,*]

1 Department of Basic Medical Sciences, Zhengzhou University, Zhengzhou 450001, China; chaofanlu@stu.zzu.edu.cn (C.-F.L.); 852436038@qq.com (S.-H.W.); summer_pxj@163.com (X.-J.P.); Hollyly1@outlook.com (H.-L.L.); 15318989671@163.com (Q.-R.L.); 15318989671@163.com @163.com (Q.-Y.L.); 18838078036@163.com (Y.-F.G.); QQ2743757146@163.com (Z.-Y.M.); Agile321@163.com (M.-J.J.); 202012402016083@gs.zzu.edu.cn(Y-R.L.)

2 Department of Pharmaceutical Sciences, Institute of Drug Discovery & Development, Key Laboratory of Advanced Drug Preparation Technologies (Ministry of Education), Zhengzhou University,
Zhengzhou 450001, China; 17671140381@sina.cn (T.Z.); zhangyb@zzu.edu.cn (Y.-B.Z.)

3 Henan Institute of Advanced Technology, Zhengzhou University, Zhengzhou 450001, China

4 Faculty of Science, The University of Melbourne, Melbourne VIC 3010, Australia

* Correspondence: mumuandzz@163.com (J.S.); saiyangz@zzu.edu.cn (S.-Y.Z.)

† These authors contributed equally to this work.

- ^{1}H NMR of Compound **13a**

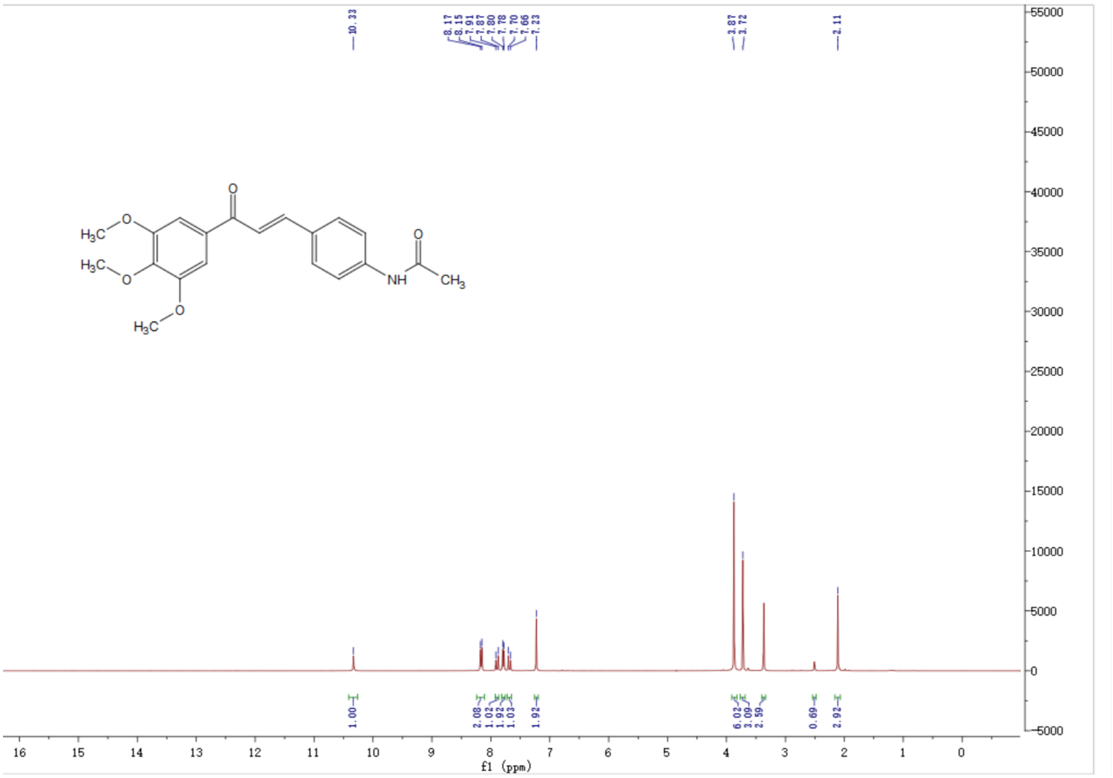

Figure S1. ^{1}H NMR spectrum of compound **13a** (400 MHz, DMSO-d_6)

- ^{13}C-NMR of Compound **13a**

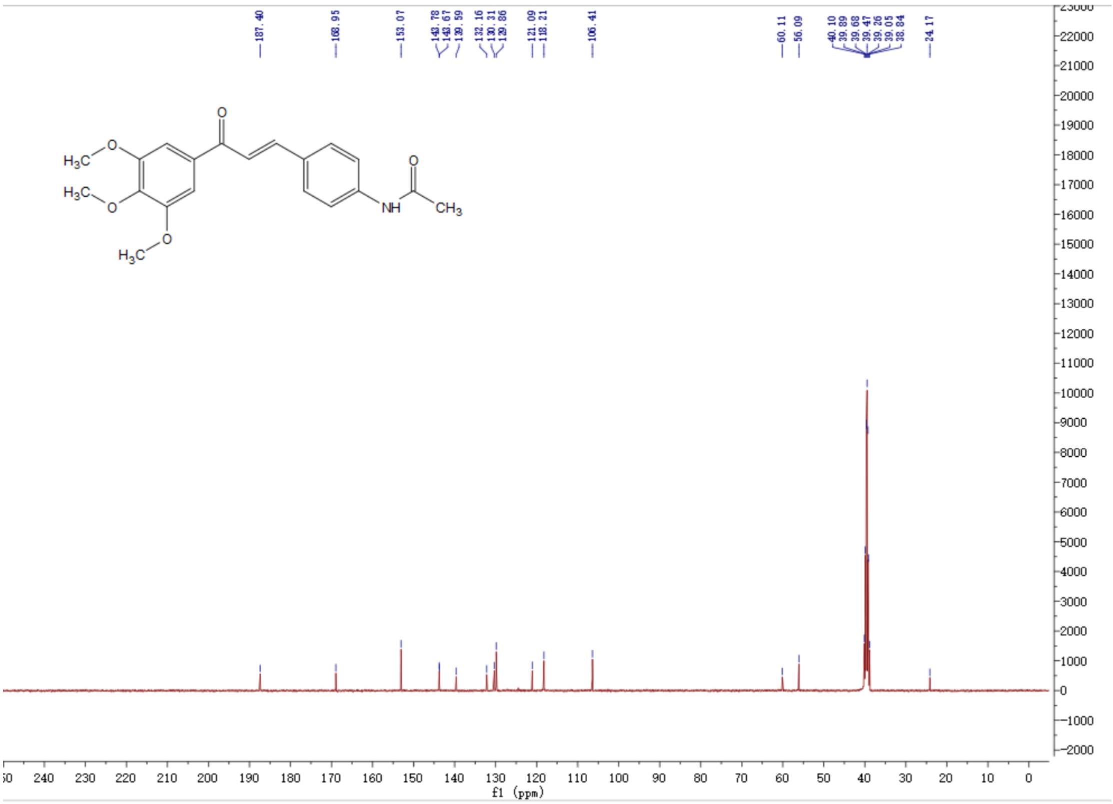

Figure 2. ^{13}C NMR spectrum of compound **13a** (100 MHz, DMSO-d_6).

- HRMS of Compound **13a**

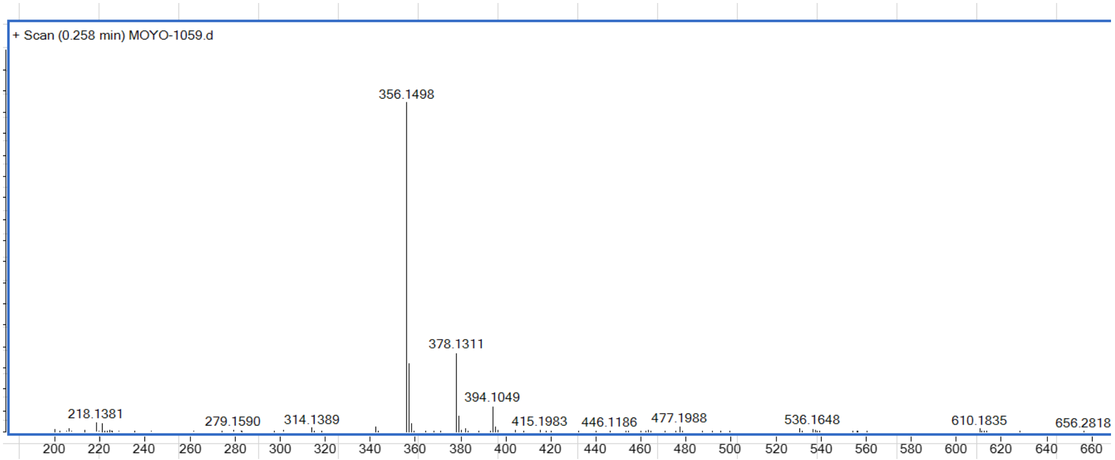

Figure S3. HRMS spectrum of compound **13a**

- ^{1}H NMR of Compound **13b**

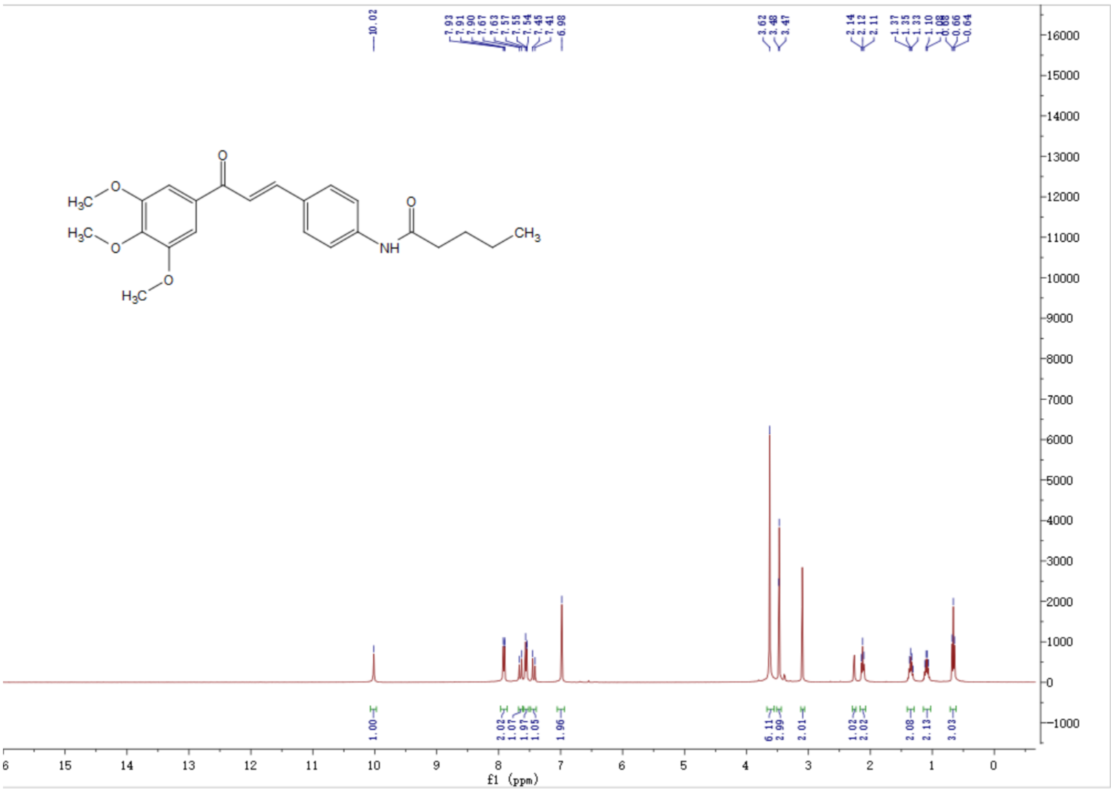

Figure S4. ^{1}H NMR spectrum of compound **13b** (400 MHz, DMSO-*d6*)

- ^{13}C-NMR of Compound **13b**

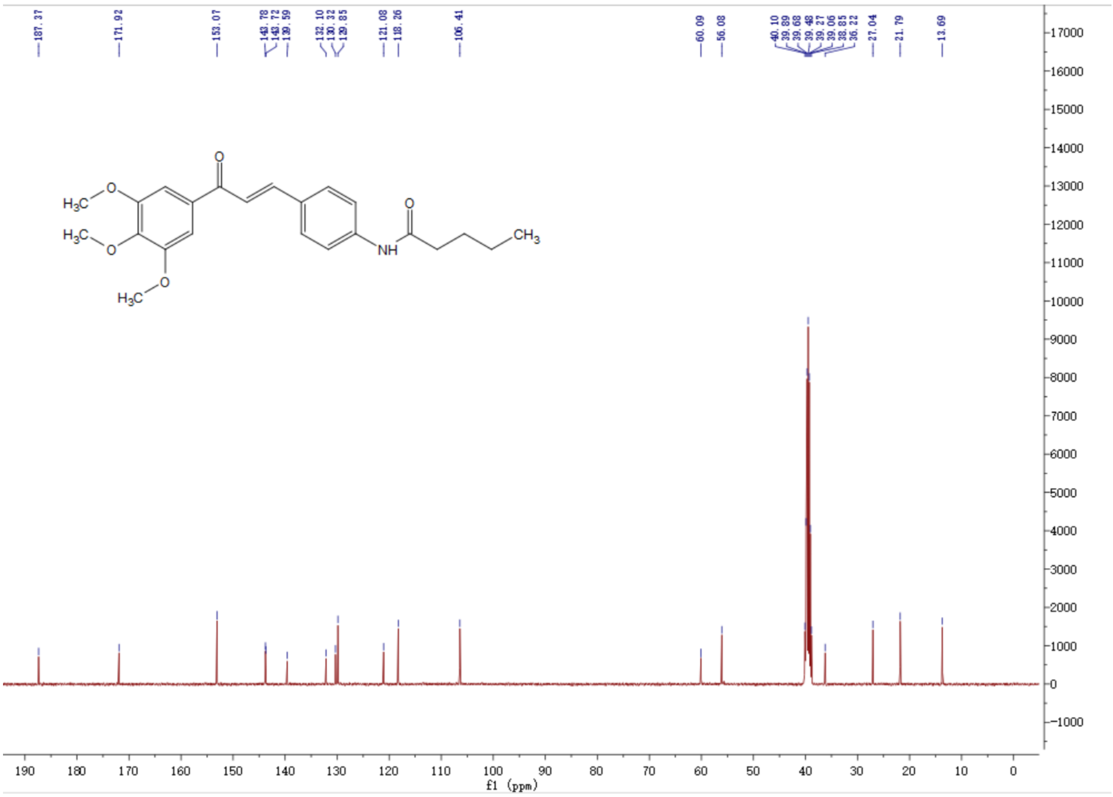

Figure S5. ^{13}C NMR spectrum of compound **13b** (100 MHz, DMSO-*d6*)

- HRMS of Compound **13b**

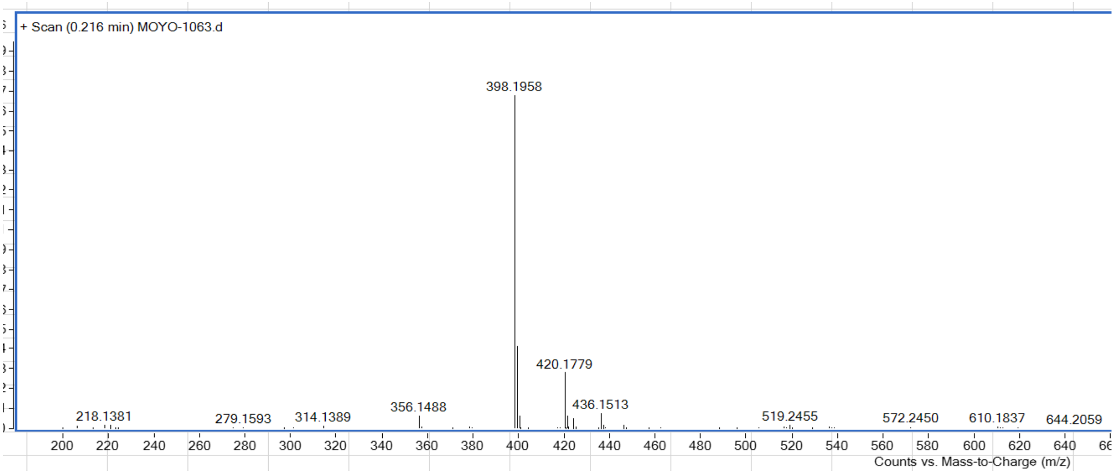

Figure S6. HRMS spectrum of compound **13b**

- ^{1}H NMR of Compound **13c**

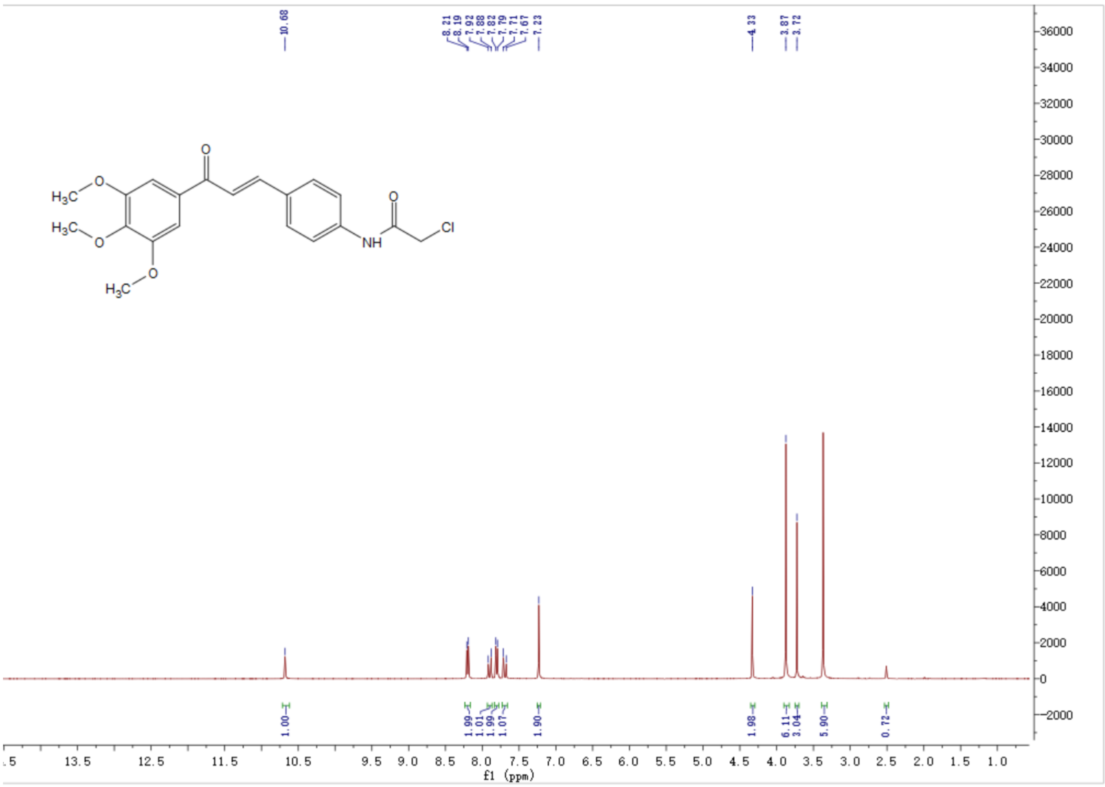

Figure S7. ^{1}H NMR spectrum of compound **13c** (400 MHz, DMSO-*d6*)

- ^{13}C-NMR of Compound **13c**

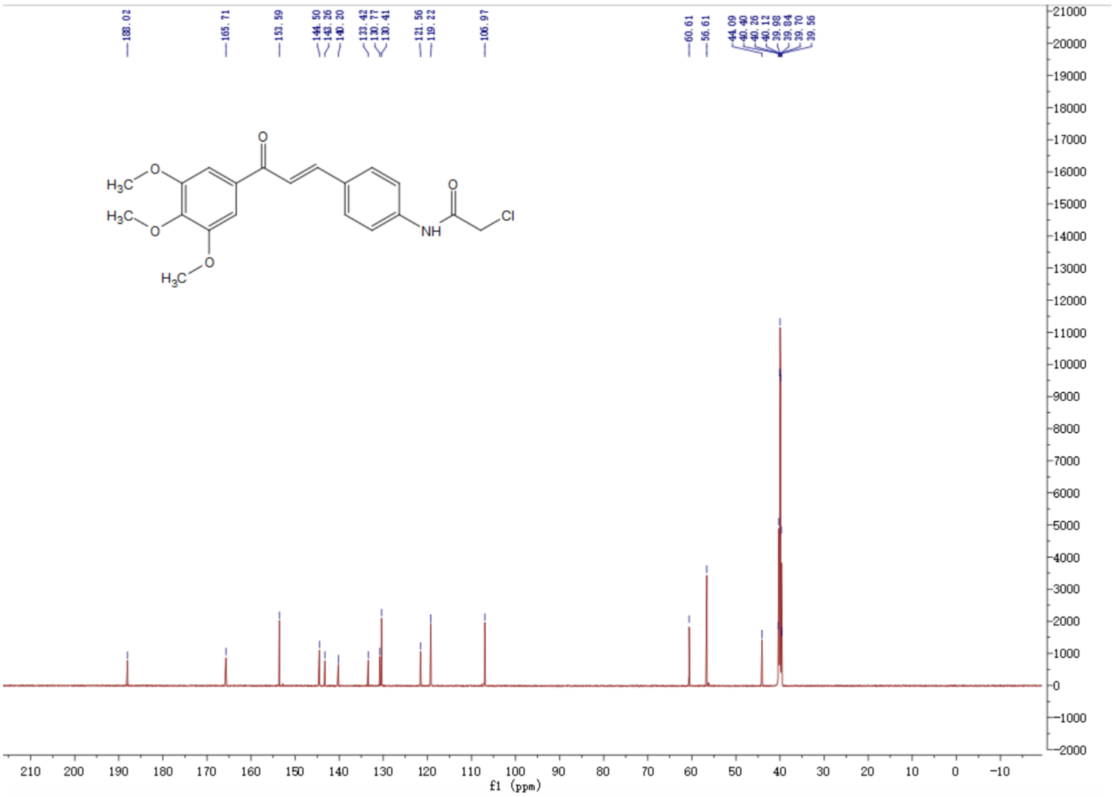

Figure S8. ^{13}C NMR spectrum of compound **13b** (151 MHz, DMSO-*d*$_6$)

- HRMS of Compound **13c**

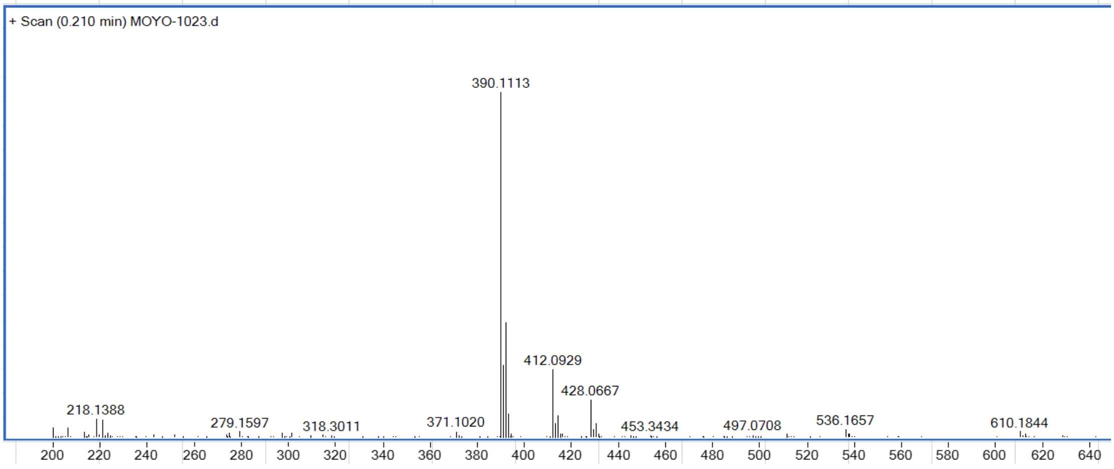

Figure S9. HRMS spectrum of compound **13c**

- ^{1}H NMR of Compound **13d**

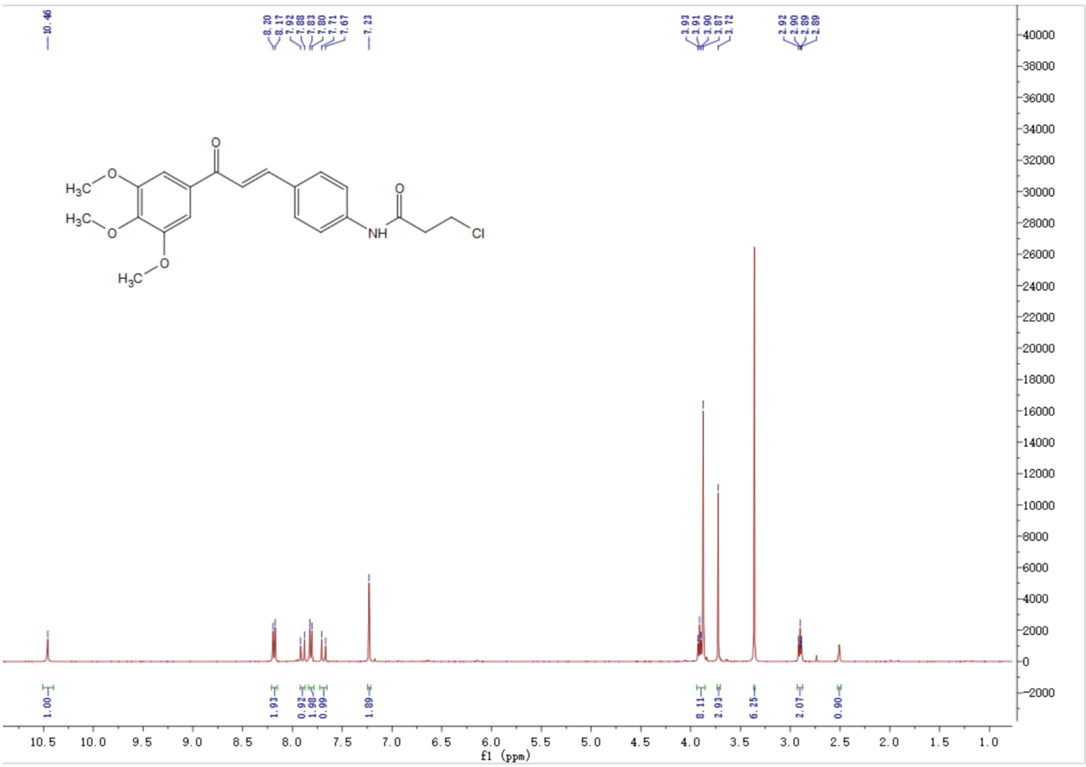

Figure S10. ^{1}H NMR spectrum of compound **13d** (400 MHz, DMSO-d_6)

- ^{13}C-NMR of Compound **13d**

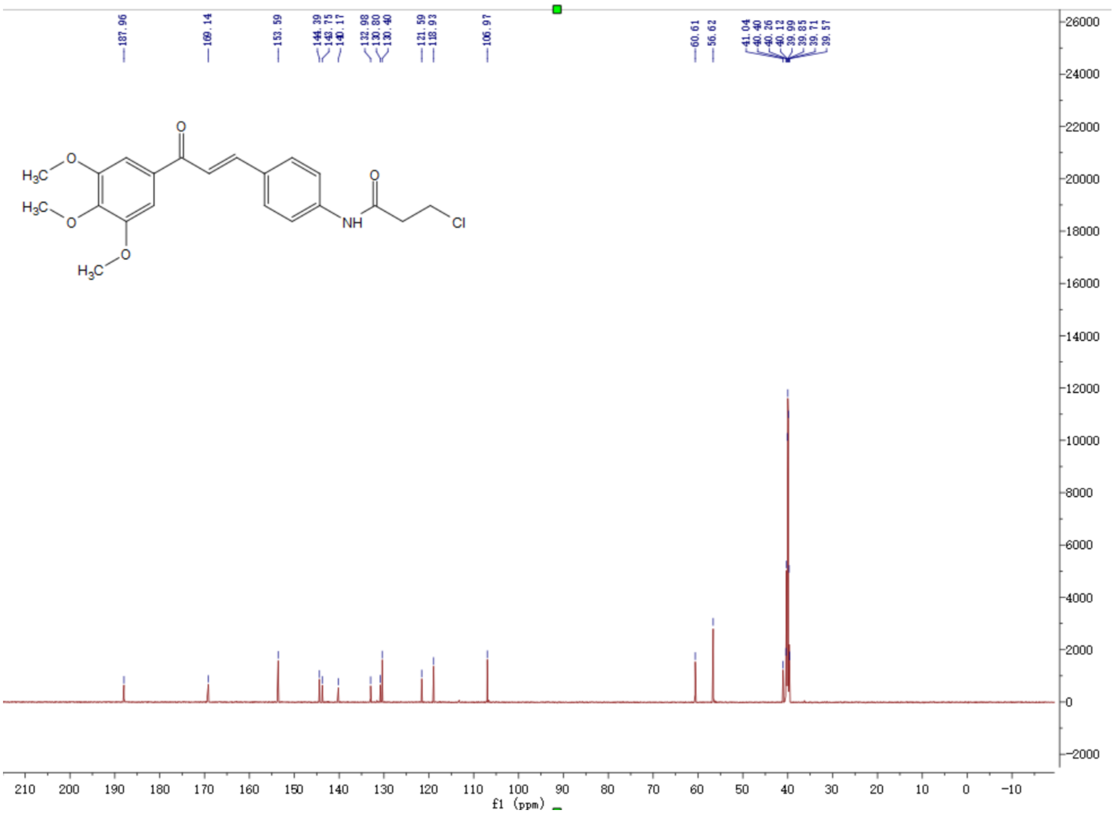

Figure S11. ^{13}C NMR spectrum of compound **13d** (151 MHz, DMSO-d_6)

- HRMS of Compound **13d**

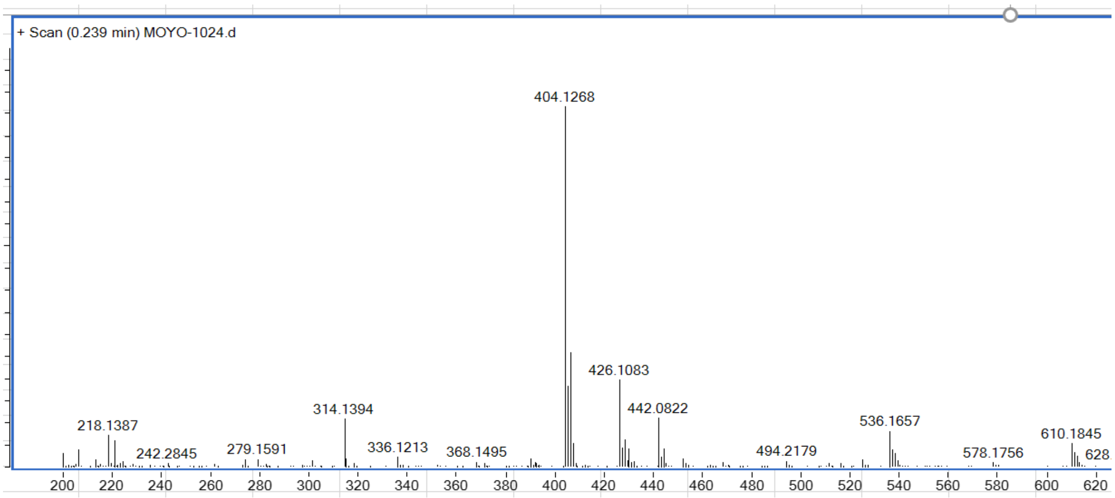

Figure S12. HRMS spectrum of compound **13d**

- ^{1}H NMR of Compound **13e**

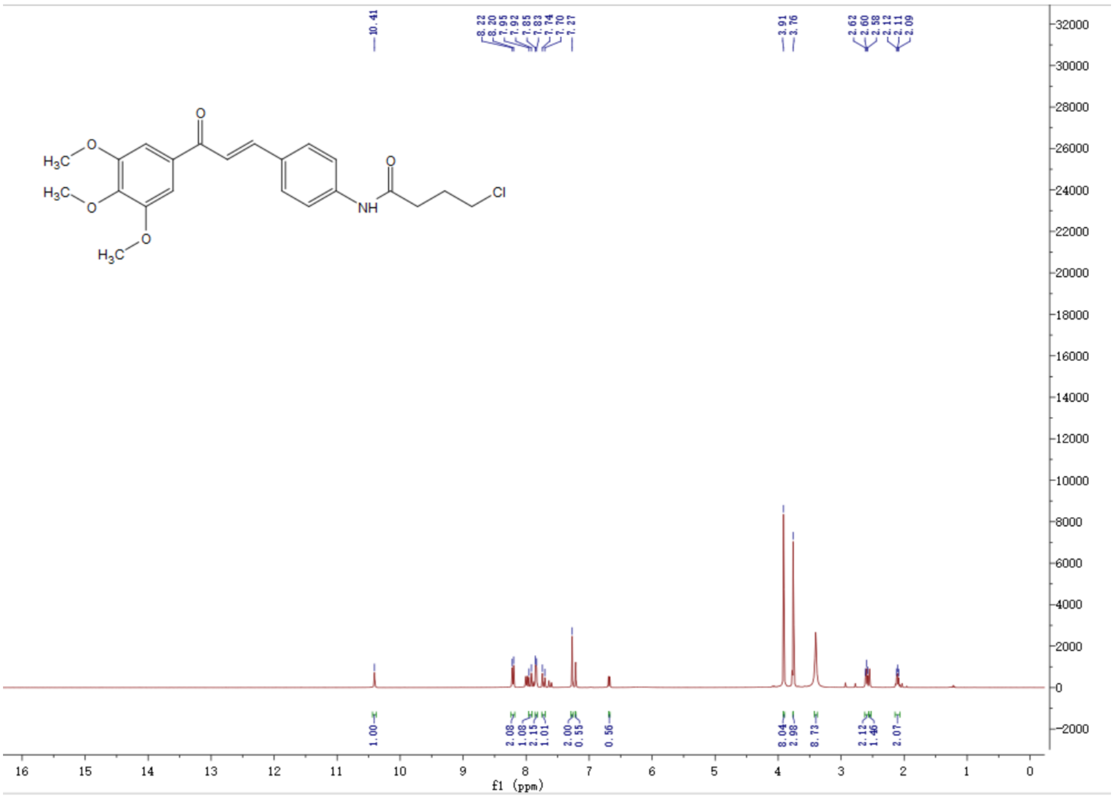

Figure S13. ^{1}H NMR spectrum of compound **13e** (400 MHz, DMSO-*d*6)

- ^{13}C-NMR of Compound **13e**

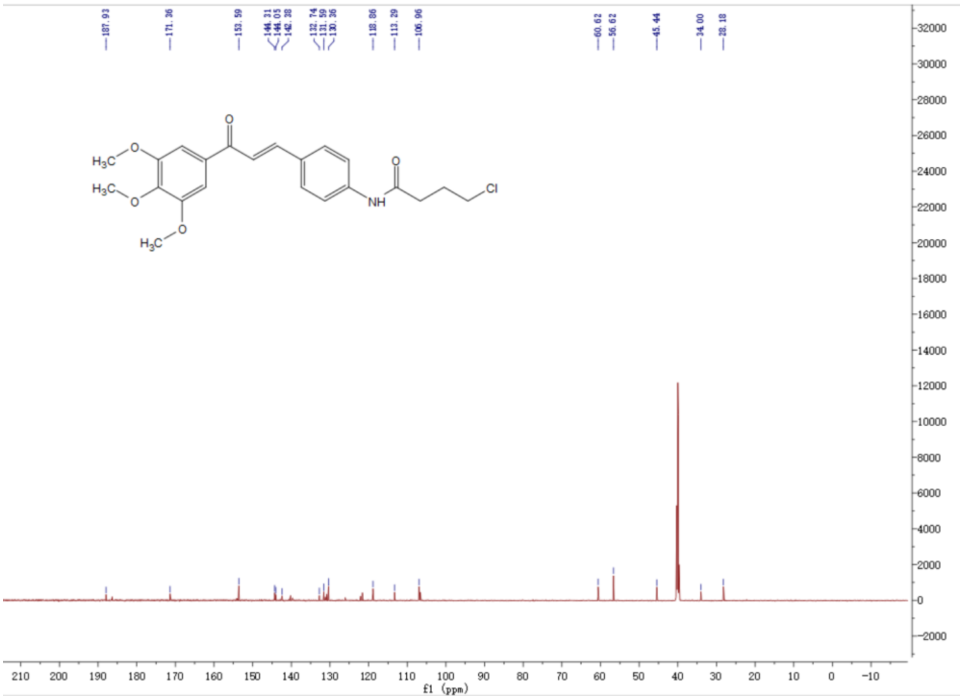

Figure S14. ^{13}C NMR spectrum of compound **13e** (151 MHz, DMSO-*d*6)

- HRMS of Compound **13e**

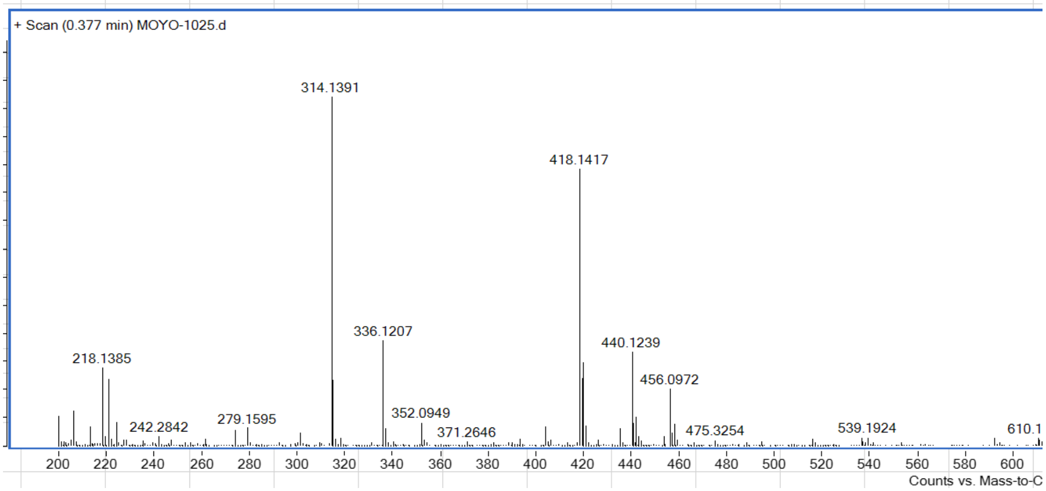

Figure S15. HRMS spectrum of compound **13e**

- ^{1}H NMR of Compound **13f**

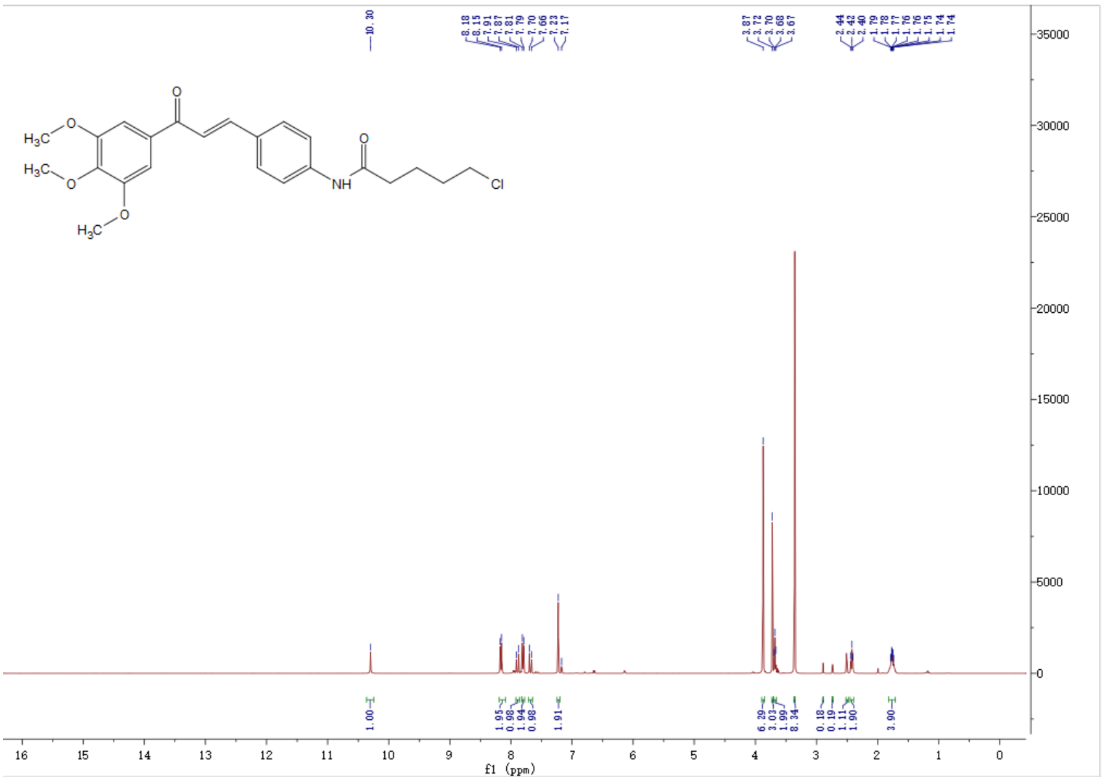

Figure S16. ^{1}H NMR spectrum of compound **13f** (400 MHz, DMSO-*d6*)

- ^{13}C-NMR of Compound **13f**

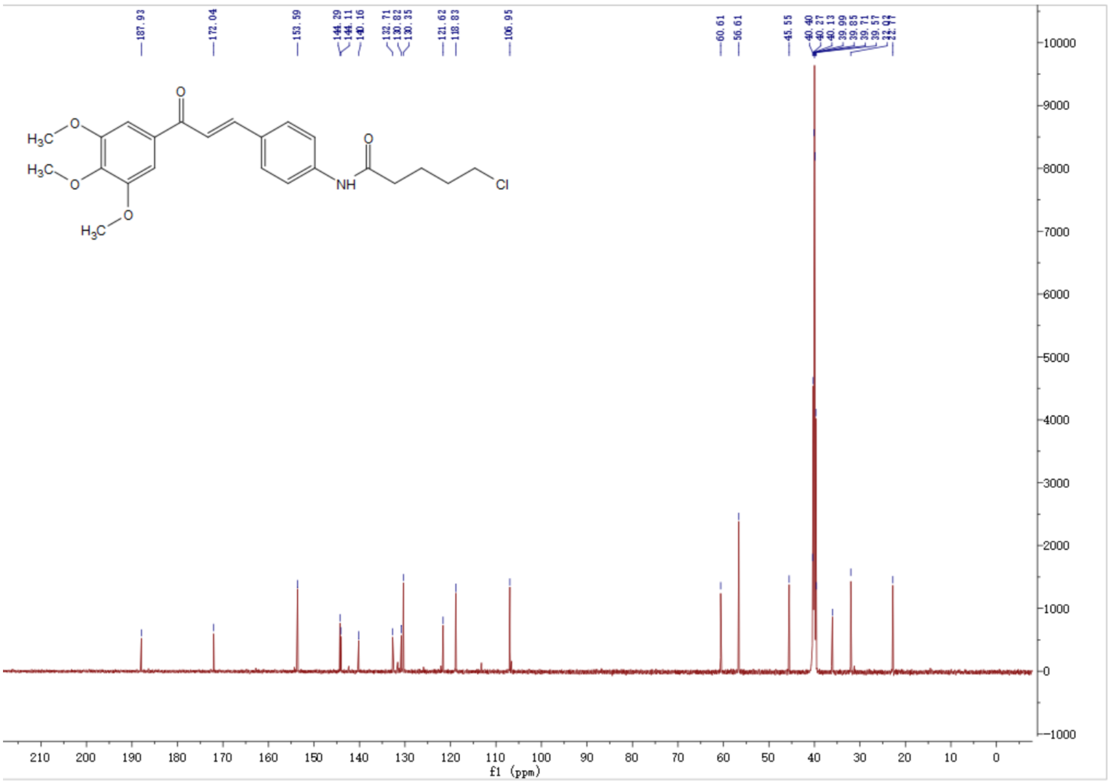

Figure S17. ^{13}C NMR spectrum of compound **13f** (151 MHz, DMSO-*d*6)

- HRMS of Compound **13f**

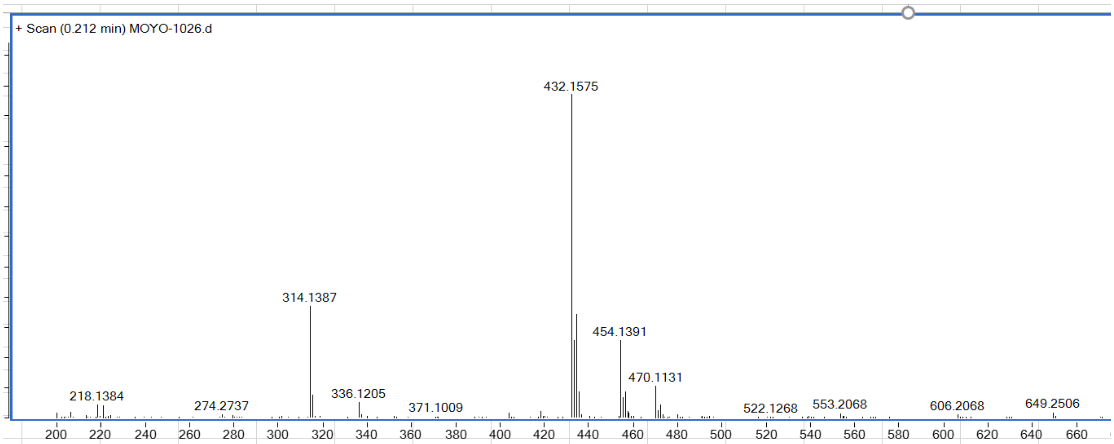

Figure S18. HRMS spectrum of compound **13f**

- ^{1}H NMR of Compound **13g**

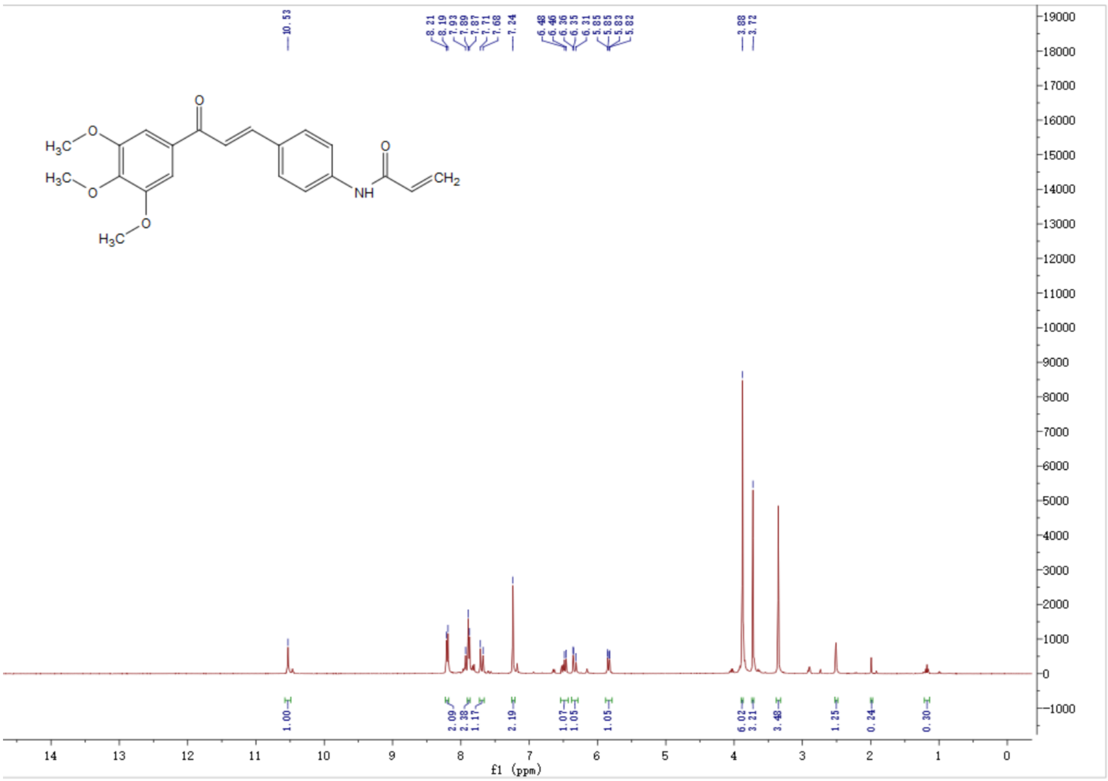

Figure S19. ^{1}H NMR spectrum of compound **13g** (400 MHz, DMSO-*d*6)

- ^{13}C-NMR of Compound **13g**

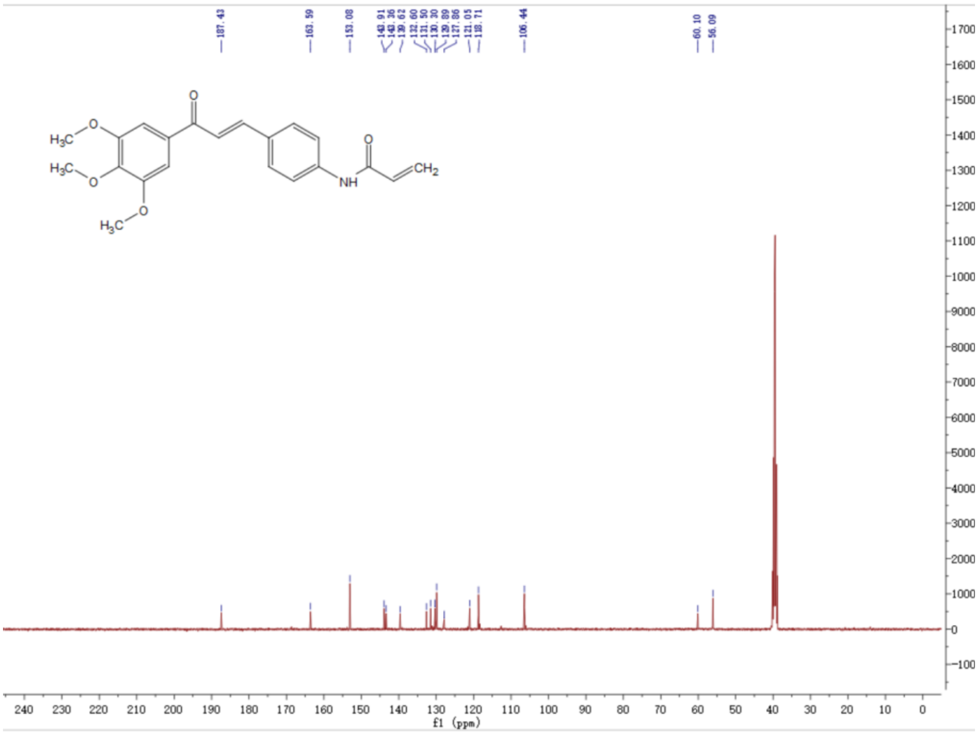

Figure S20. ^{13}C NMR spectrum of compound **13g** (100 MHz, DMSO-*d*6)

- HRMS of Compound **13g**

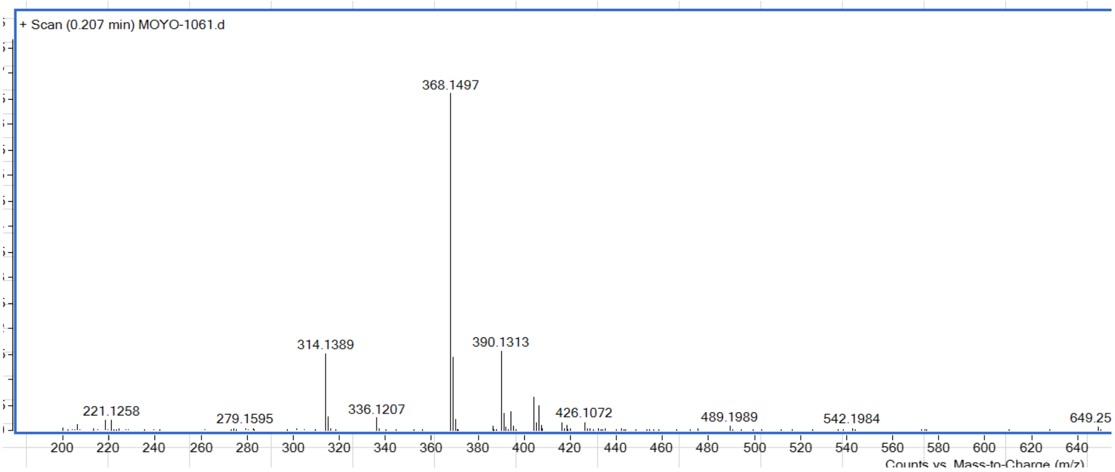

Figure S21. HRMS spectrum of compound **13g**

- ^{1}H NMR of Compound **13h**

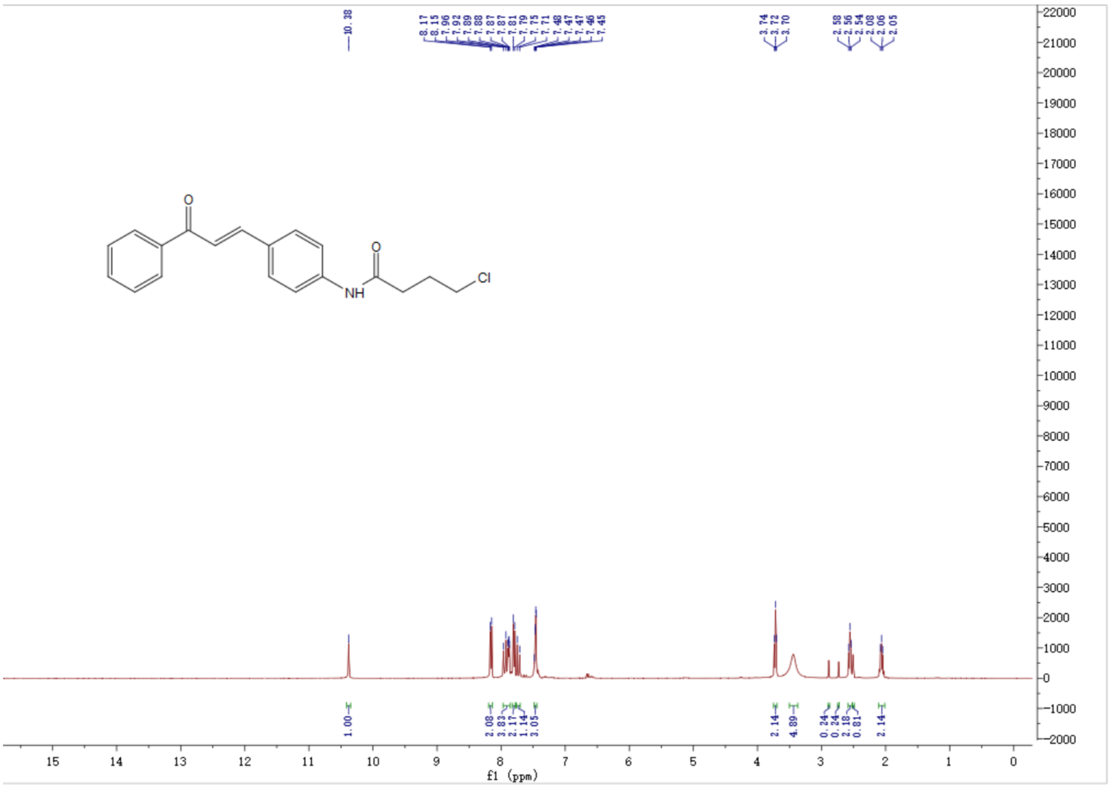

Figure S22. ^{1}H NMR spectrum of compound **13h** (400 MHz, DMSO-*d6*)

- ^{13}C-NMR of Compound **13h**

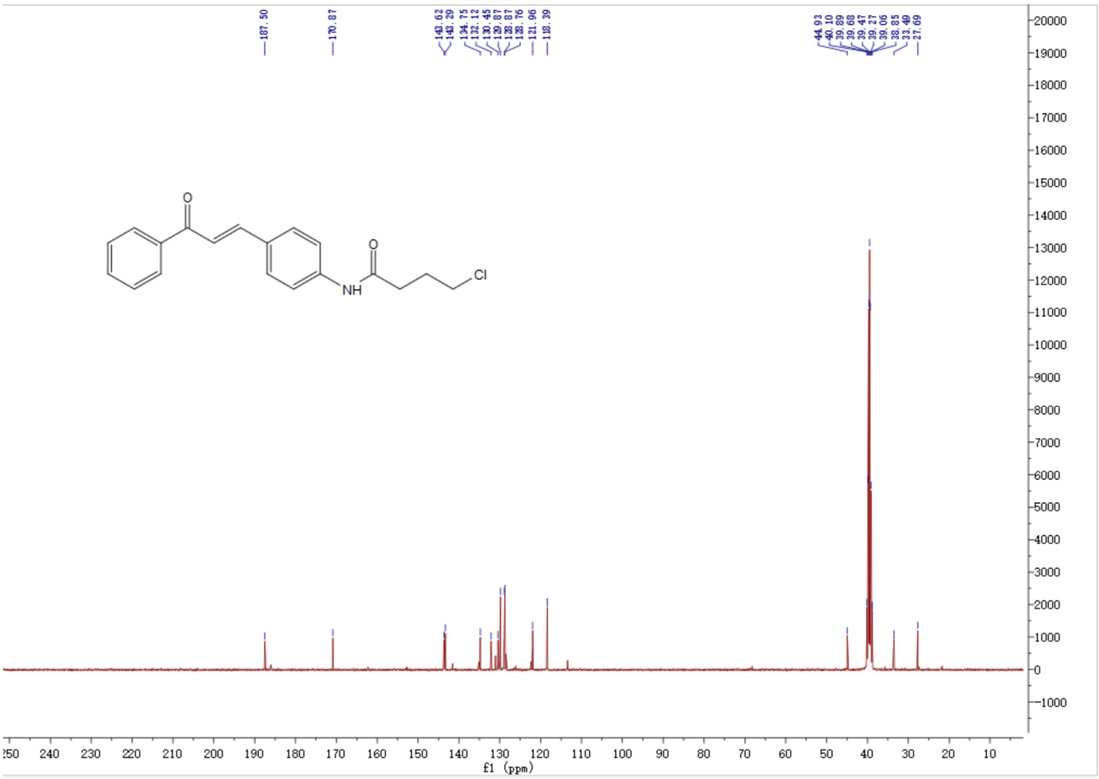

Figure S23. ^{13}C NMR spectrum of compound **13h** (100 MHz, DMSO-*d6*)

- ^{1}H NMR of Compound **13i**

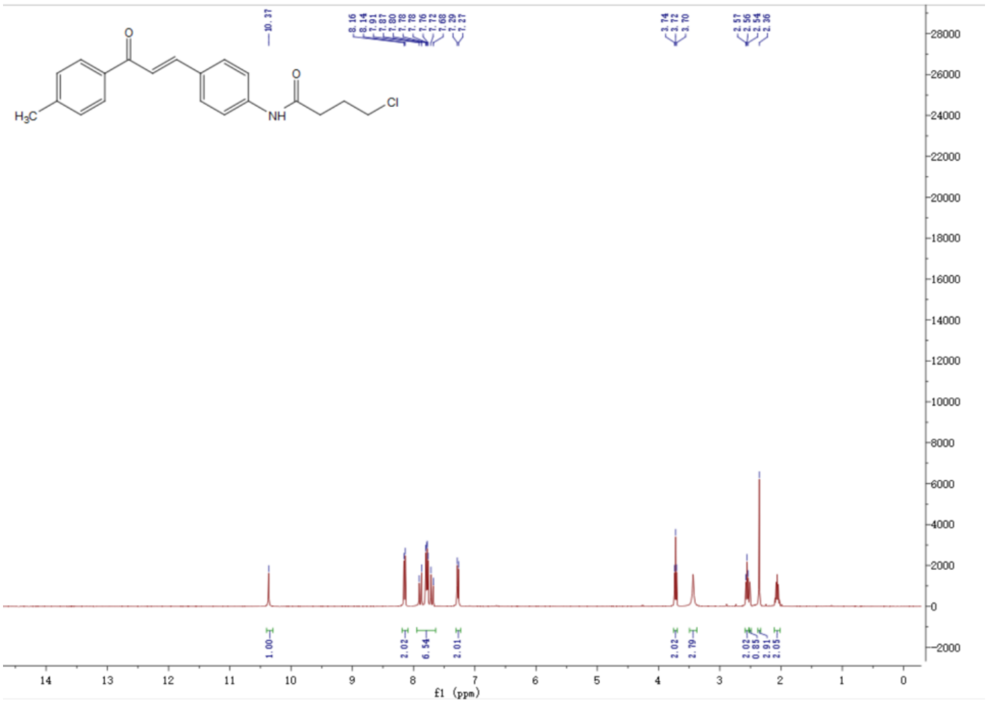

Figure S24. ^{1}H NMR spectrum of compound **13i** (400 MHz, DMSO-*d6*)

- ^{13}C-NMR of Compound **13i**

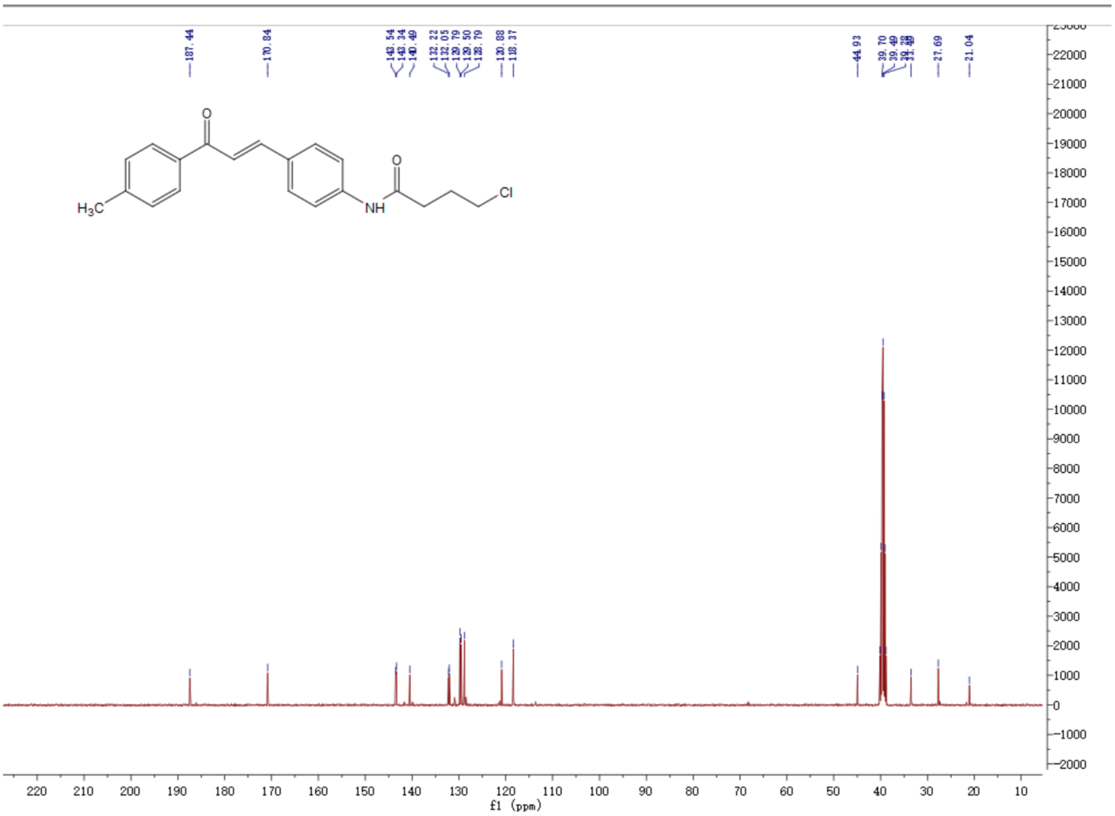

Figure S25. ^{13}C NMR spectrum of compound **13i** (100 MHz, DMSO-*d*$_6$)

- HRMS of Compound **13i**

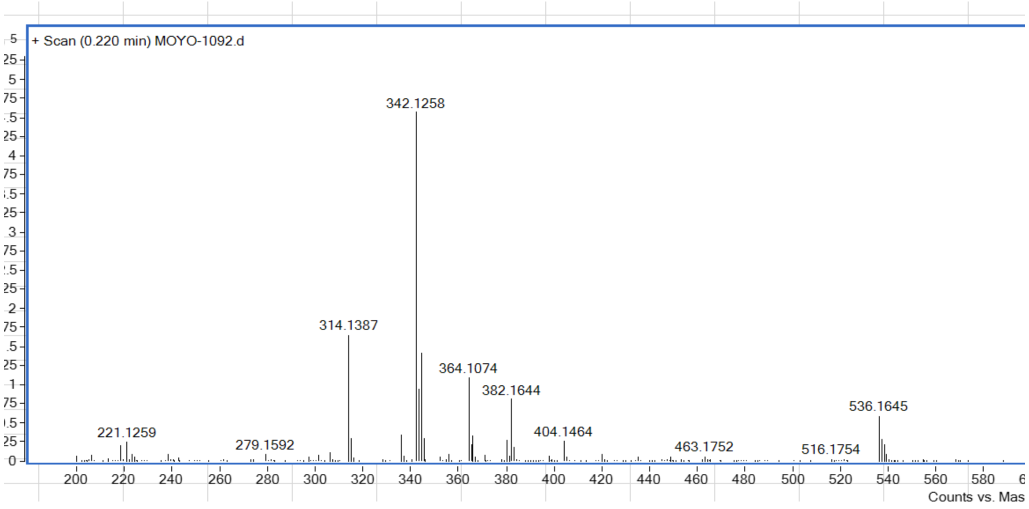

Figure S26. HRMS spectrum of compound **13i**

- ^{1}H NMR of Compound **13j**

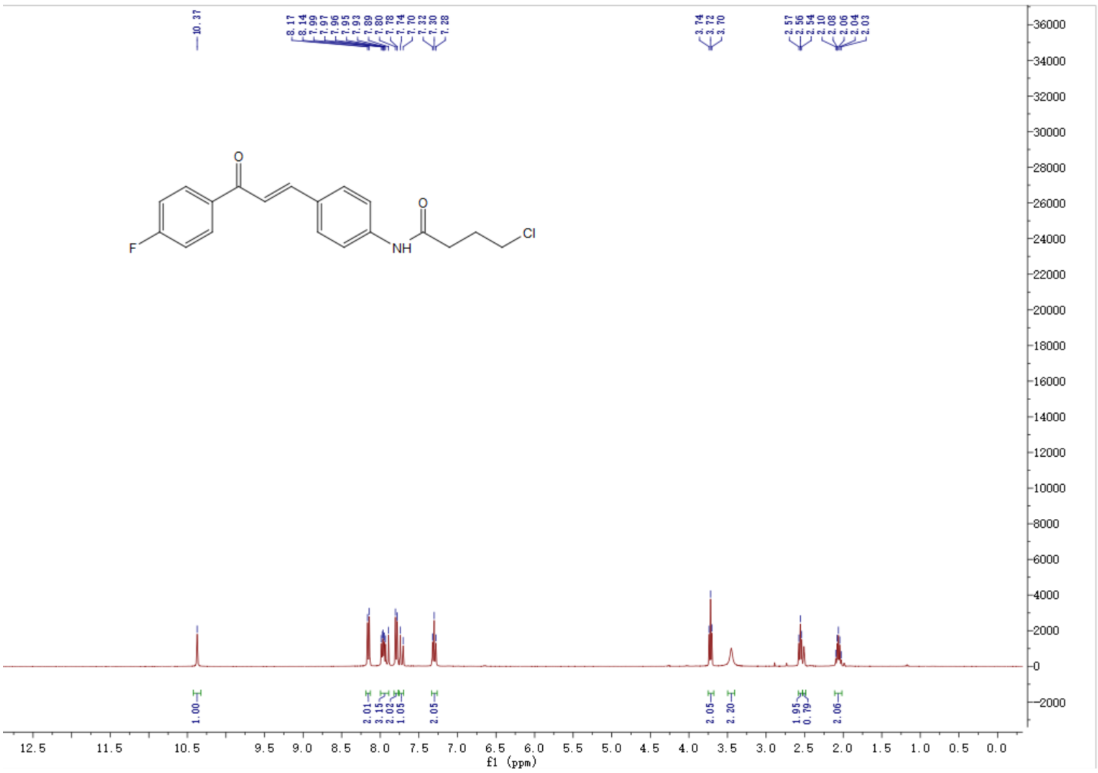

Figure S27. ^{1}H NMR spectrum of compound **13j** (400 MHz, DMSO-d_6)

- ^{13}C-NMR of Compound **13j**

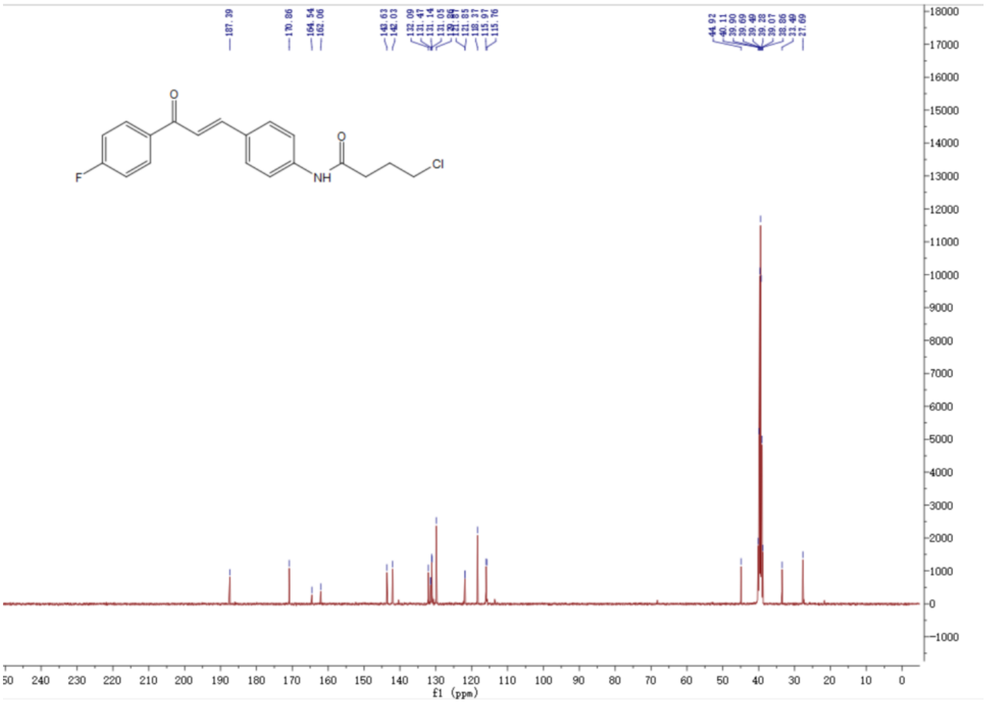

Figure S28. ^{13}C NMR spectrum of compound **13j** (100 MHz, DMSO-d_6)

- ^{1}H NMR of Compound **13j**

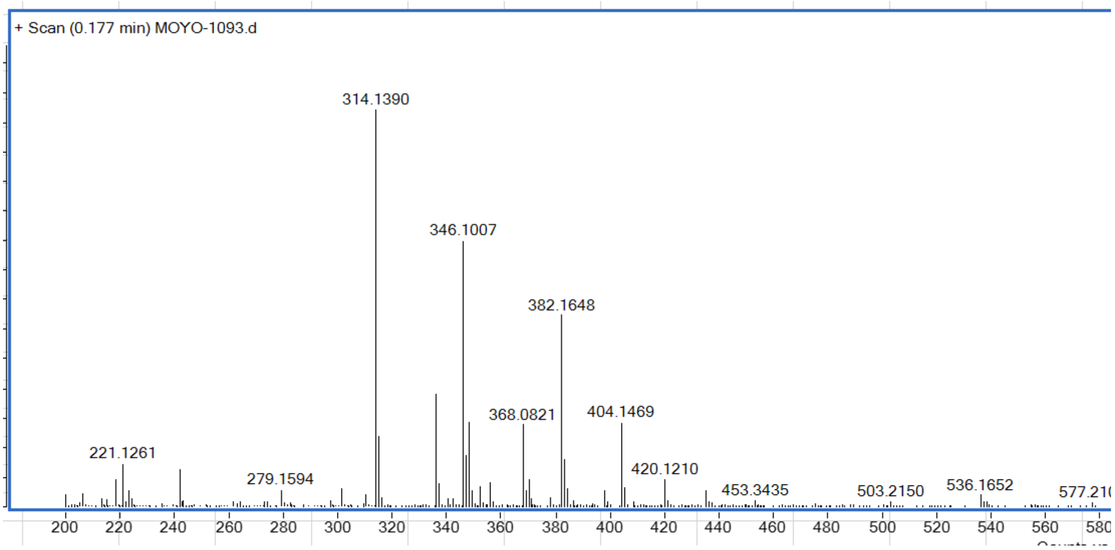

Figure S29. HRMS spectrum of compound **13j**

- ^{1}H NMR of Compound **13k**

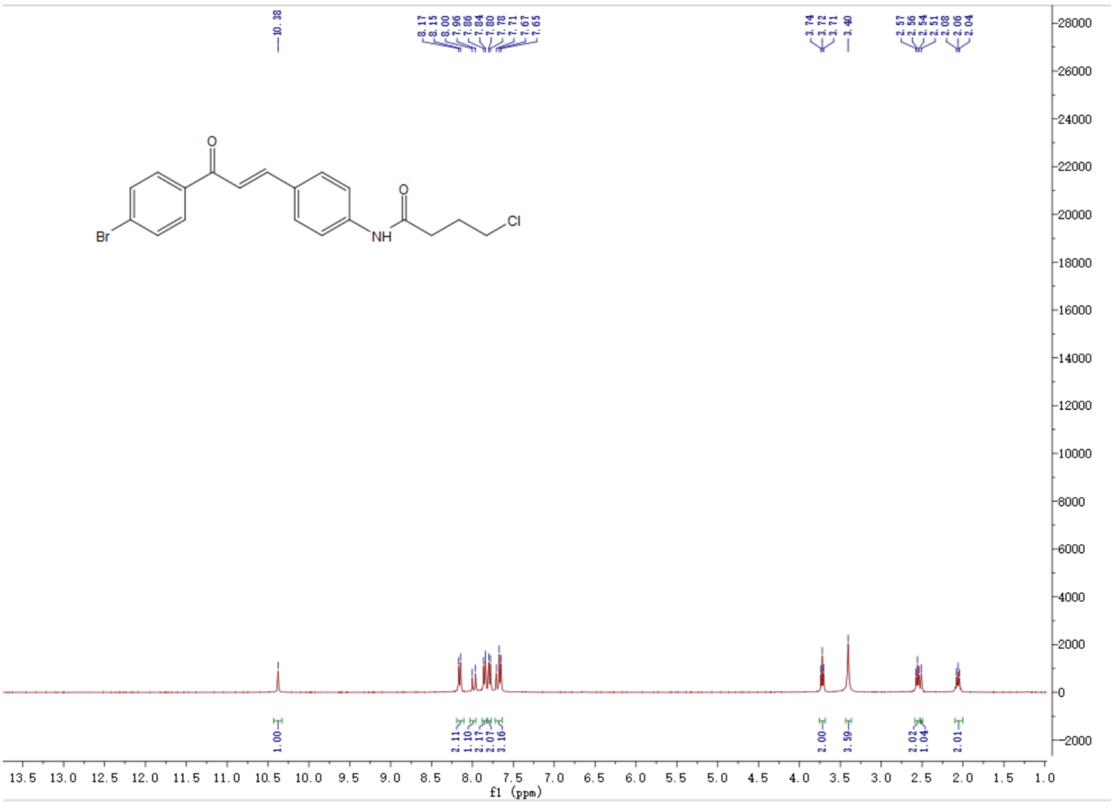

Figure S30. ^{1}H NMR spectrum of compound **13k** (400 MHz, DMSO-d_6)

- ^{13}C-NMR of Compound **13k**

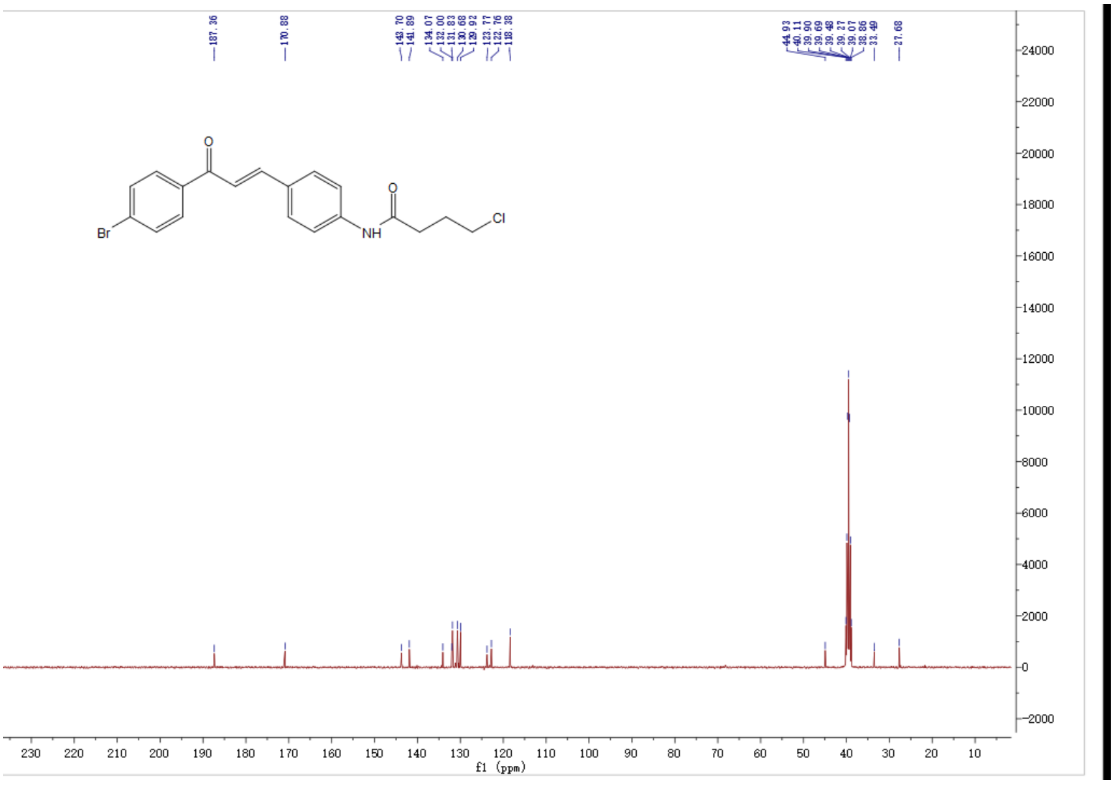

Figure S31. ^{13}C NMR spectrum of compound **13k** (100 MHz, DMSO-d_6)

- HRMS of Compound **13k**

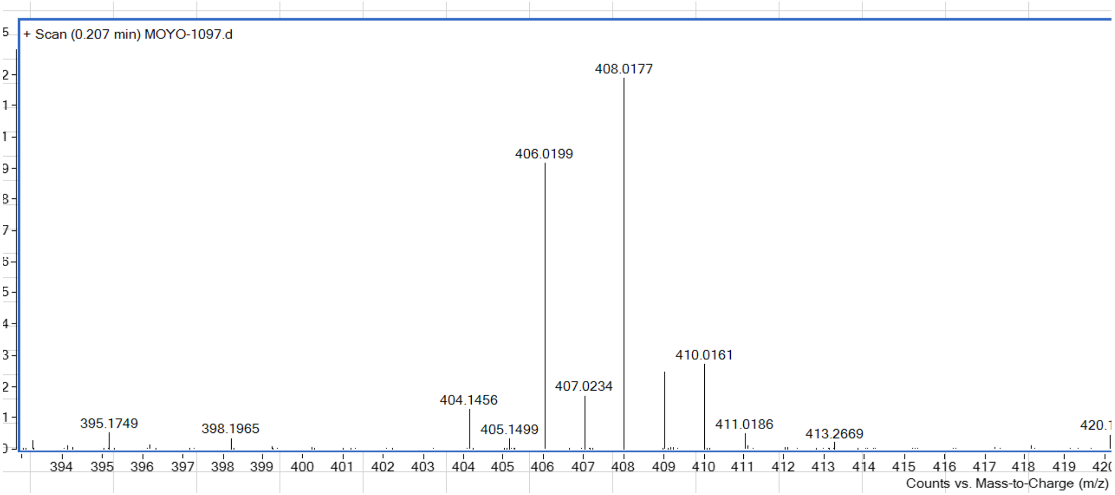

Figure S32. HRMS spectrum of compound **13k**

- ^{1}H NMR of Compound **13l**

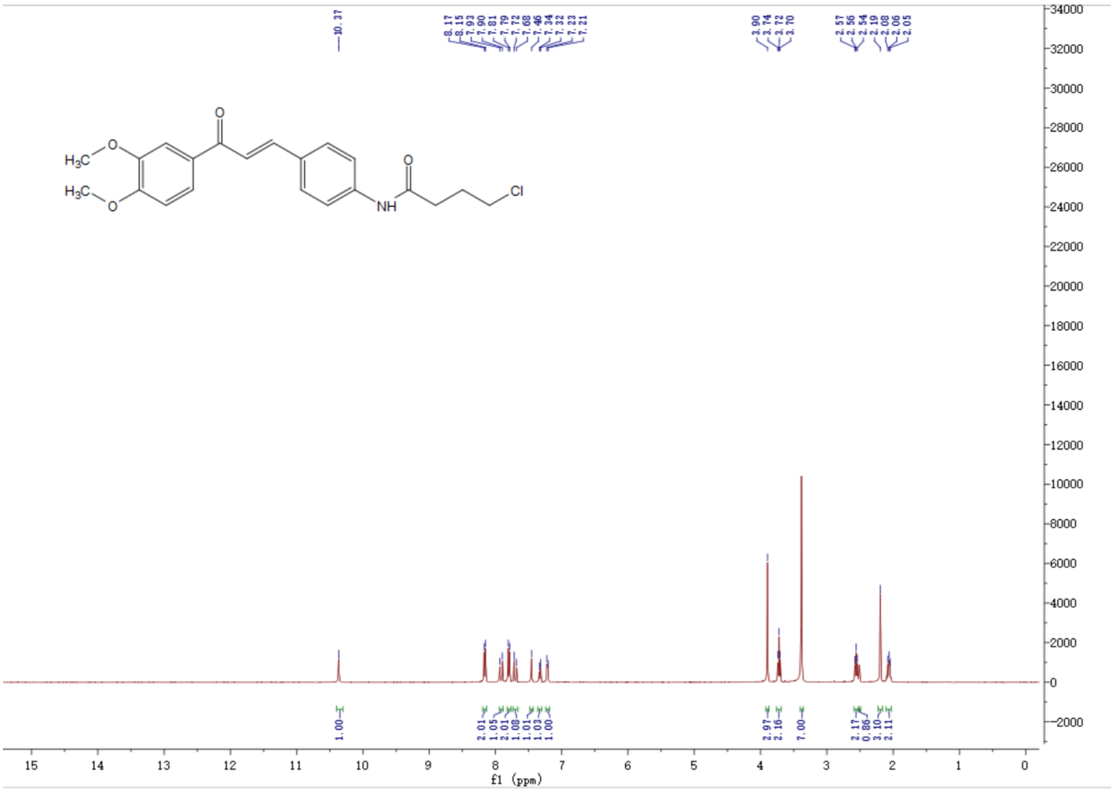

Figure S33. ^{1}H NMR spectrum of compound **13l** (400 MHz, DMSO-*d*6)

- ^{13}C-NMR of Compound **13l**

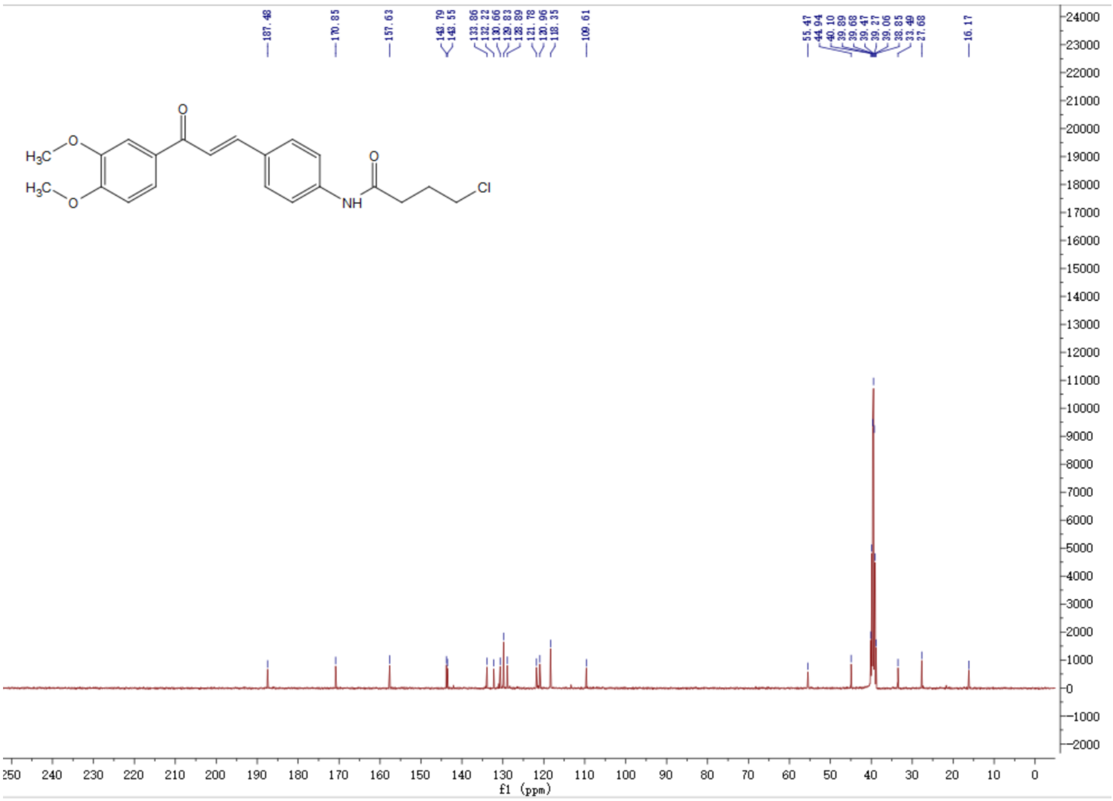

Figure S34. ^{13}C NMR spectrum of compound **13l** (100 MHz, DMSO-d_6)

- HRMS of Compound **13l**

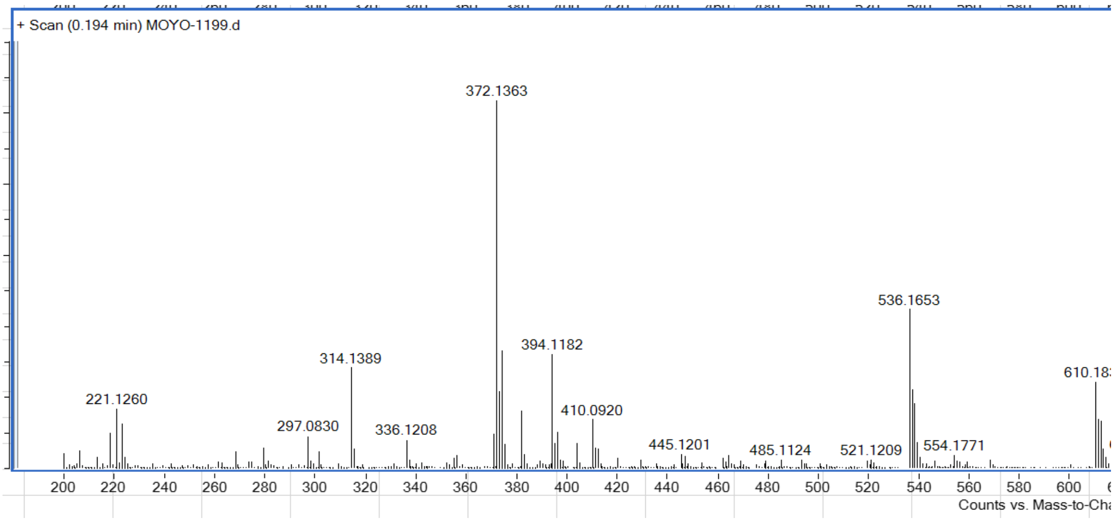

Figure S35. HRMS spectrum of compound **13l**

- ^{1}H NMR of Compound **13m**

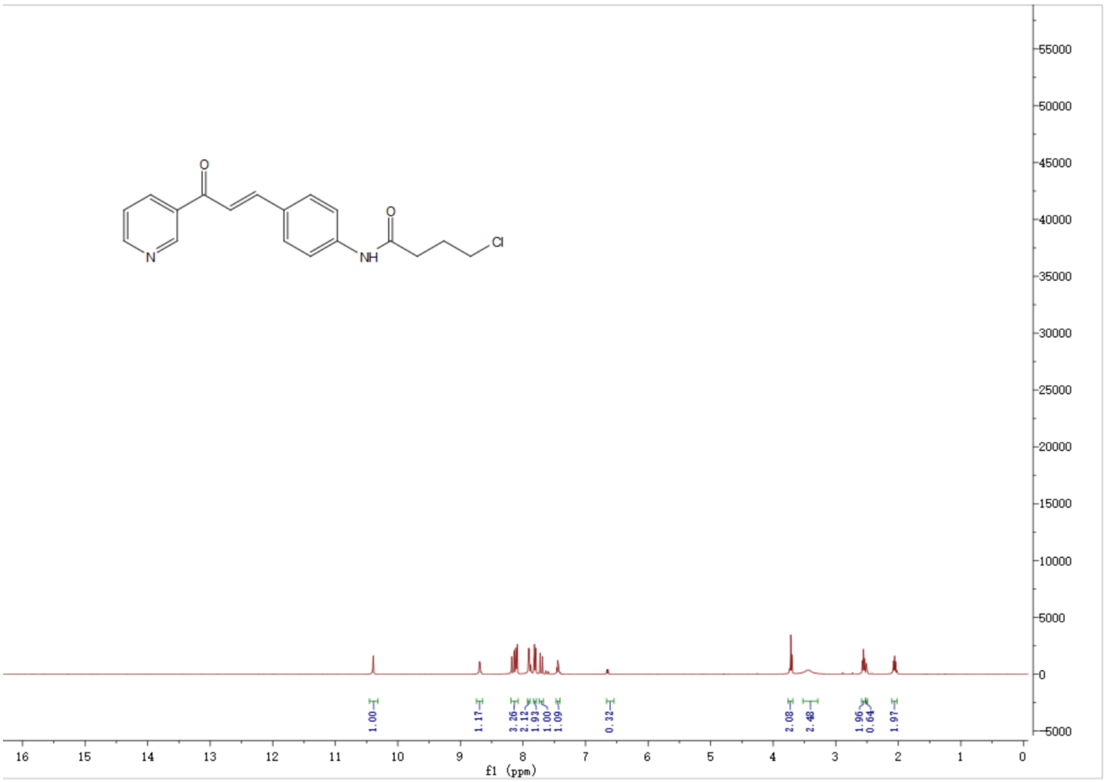

Figure S36. ^{1}H NMR spectrum of compound **13m** (400 MHz, DMSO-d_6)

- ^{13}C-NMR of Compound **13m**

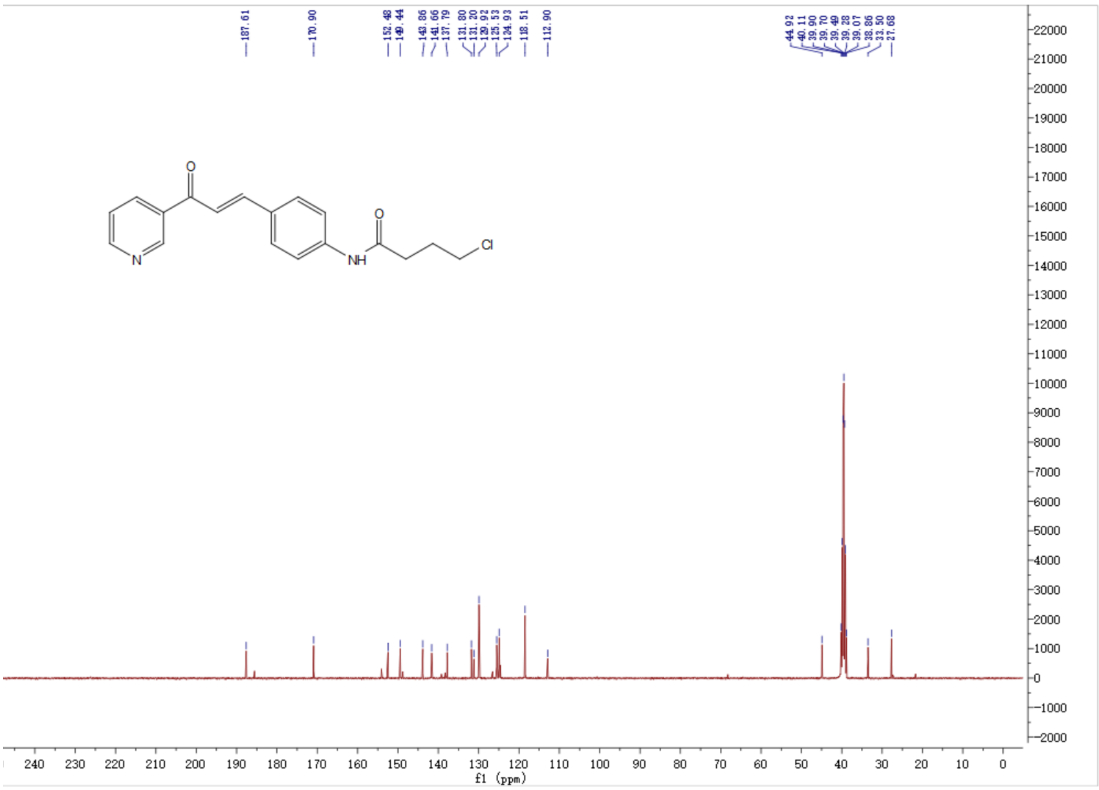

Figure S37. ^{13}C NMR spectrum of compound **13m** (100 MHz, DMSO-d_6)

- HRMS of Compound **13m**

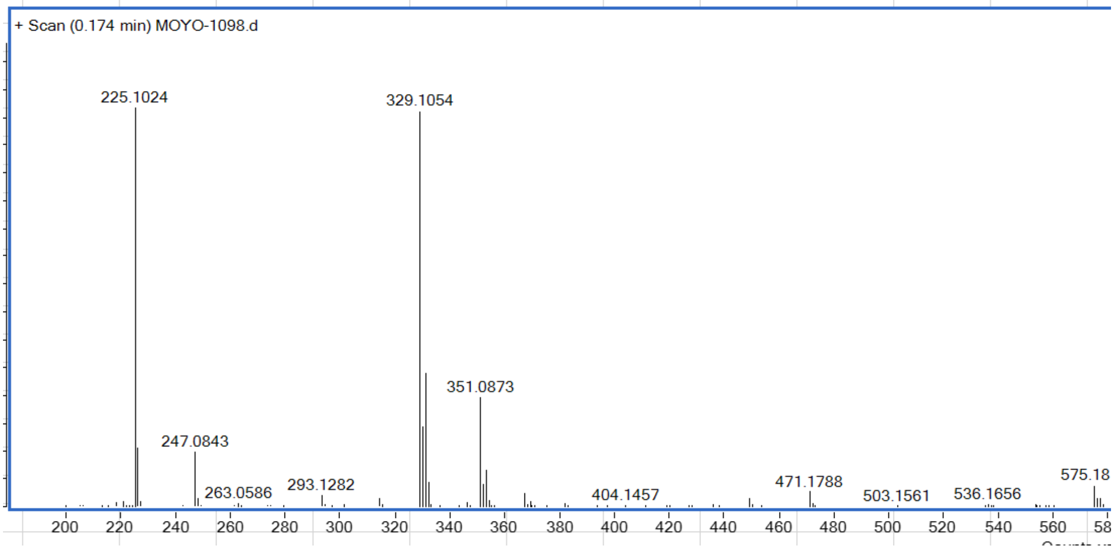

Figure S38. HRMS spectrum of compound **13m**

- ^{1}H NMR of Compound **13n**

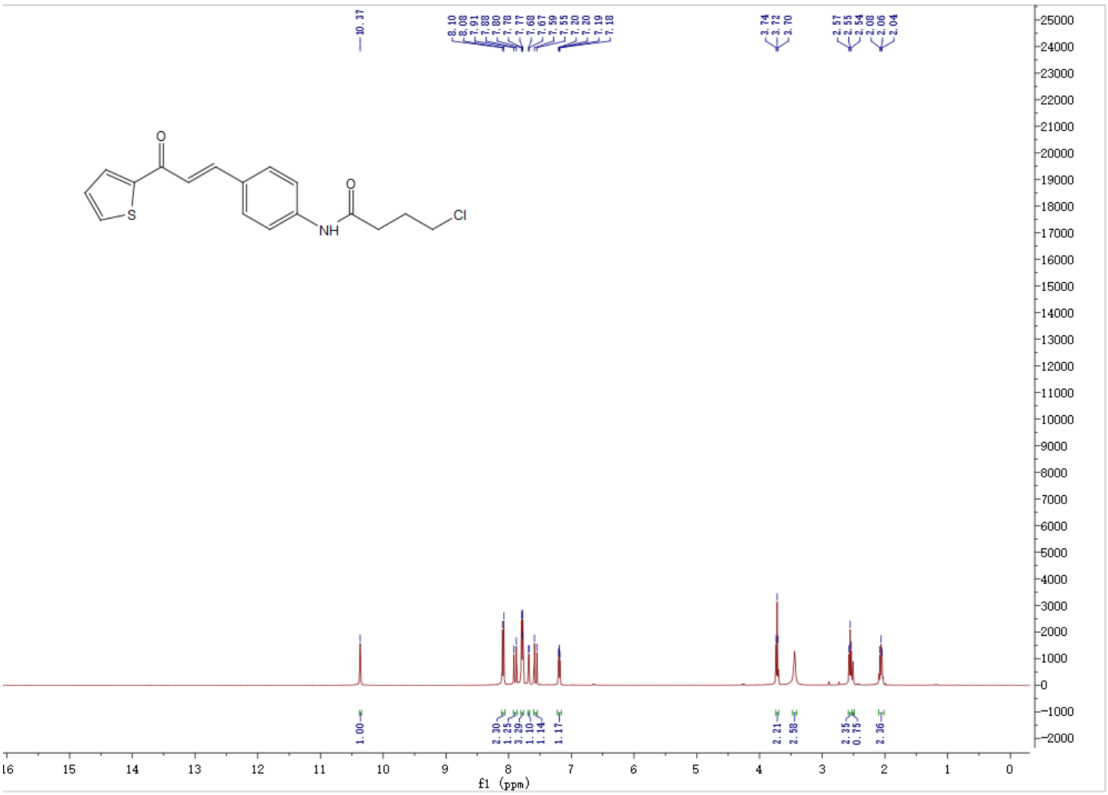

Figure S39. ^{1}H NMR spectrum of compound **13n** (400 MHz, DMSO-d_6)

- ^{13}C-NMR of Compound **13n**

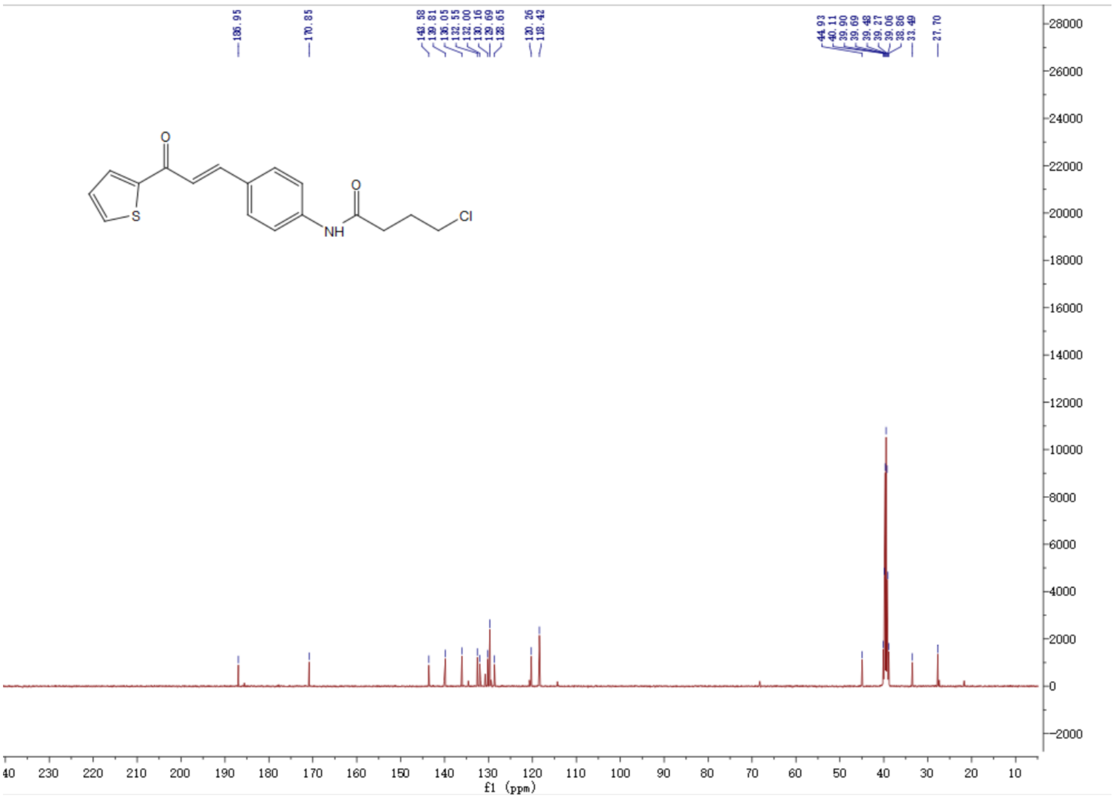

Figure S40. ^{13}C NMR spectrum of compound **13n** (100 MHz, DMSO-d_6)

- HRMS of Compound **13n**

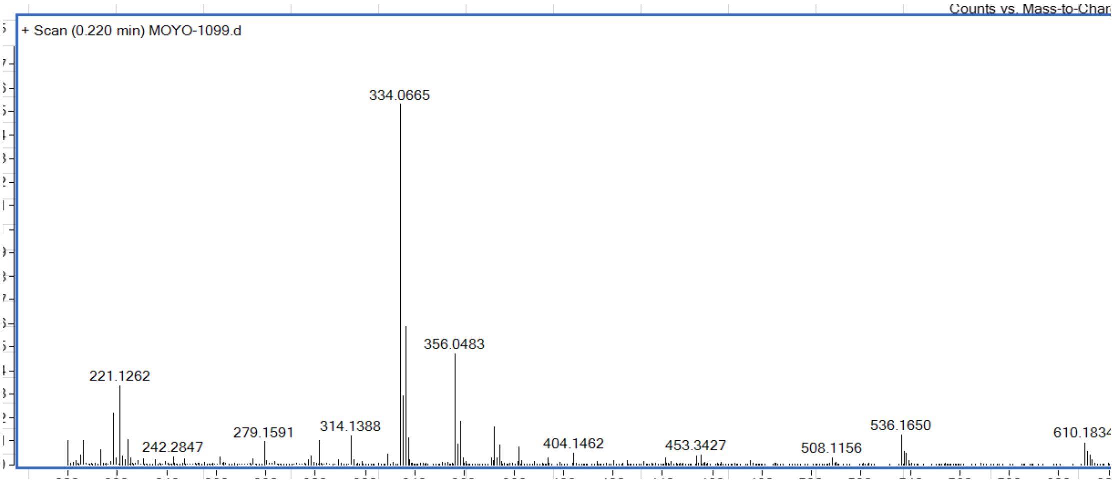

Figure S41. HRMS spectrum of compound **13n**
